# *Pseudomonas aeruginosa* inhibits the growth of *Scedosporium aurantiacum*, an opportunistic fungal pathogen isolated from the lungs of cystic fibrosis patients

**DOI:** 10.3389/fmicb.2015.00866

**Published:** 2015-08-24

**Authors:** Jashanpreet Kaur, Bhavin P. Pethani, Sheemal Kumar, Minkyoung Kim, Anwar Sunna, Liisa Kautto, Anahit Penesyan, Ian T. Paulsen, Helena Nevalainen

**Affiliations:** ^1^Department of Chemistry and Biomolecular Sciences, Macquarie UniversitySydney, NSW, Australia; ^2^Biomolecular Frontiers Research Centre, Macquarie UniversitySydney, NSW, Australia

**Keywords:** co-culture, *S. aurantiacum*, *P. aeruginosa*, interactions, growth inhibition, phenazines, SCFM, biofilms

## Abstract

The filamentous fungus *Scedosporium aurantiacum* and the bacterium *Pseudomonas aeruginosa* are opportunistic pathogens isolated from lungs of the cystic fibrosis (CF) patients. *P. aeruginosa* has been known to suppress the growth of a number of CF related fungi such as *Aspergillus fumigatus, Candida albicans*, and *Cryptococcus neoformans*. However, the interactions between *P. aeruginosa* and *S. aurantiacum* have not been investigated in depth. Hence we assessed the effect of *P. aeruginosa* reference strain PAO1 and two clinical isolates PASS1 and PASS2 on the growth of two clinical *S. aurantiacum* isolates WM 06.482 and WM 08.202 using solid plate assays and liquid cultures, in a synthetic medium mimicking the nutrient condition in the CF sputum. Solid plate assays showed a clear inhibition of growth of both *S. aurantiacum* strains when cultured with *P. aeruginosa* strains PASS1 and PAO1. The inhibitory effect was confirmed by confocal microscopy. In addition to using chemical fluorescent stains, strains tagged with yfp (*P. aeruginosa* PASS1) and mCherry (*S. aurantiacum* WM 06.482) were created to facilitate detailed microscopic observations on strain interaction. To our knowledge, this is the first study describing successful genetic transformation of *S. aurantiacum*. Inhibition of growth was observed only in co-cultures of *P. aeruginosa* and *S. aurantiacum*; the cell fractions obtained from independent bacterial monocultures failed to initiate a response against the fungus. In the liquid co-cultures, biofilm forming *P. aeruginosa* strains PASS1 and PAO1 displayed higher inhibition of fungal growth when compared to PASS2. No change was observed in the inhibition pattern when direct cell contact between the bacterial and fungal strains was prevented using a separation membrane suggesting the involvement of extracellular metabolites in the fungal inhibition. However, one of the most commonly described bacterial virulence factors, pyocyanin, had no effect against either of the *S. aurantiacum* strains. This study shows that *P. aeruginosa* has a substantial inhibitory effect on the growth of the recently described CF fungal pathogen *S. aurantiacum*. The findings also highlighted that *P. aeruginosa* biofilm formation is important but not crucial for inhibiting the growth of *S. aurantiacum* in a lung- mimicking environment.

## Introduction

Cystic fibrosis (CF) is one of the most common, potentially lethal, genetically inherited disorders affecting mainly the European Caucasian population (O’Sullivan and Freedman, 2009). Although the disease affects a number of organs and systems in the human body, lungs remain the main site of infection in CF patients (Quinton, 1999). The inherited condition stems from the mutation of the CF transmembrane conductance regulator (CFTR) gene, which regulates the transport of chloride ions across the plasma membrane of the epithelial cells (Boucher, 2007). Impaired ion exchange reduces the mucociliary clearance, which leads to accumulation of hyper-viscous mucus in the airway surfaces, thus providing ideal conditions for the growth of microorganisms (Delhaes et al., 2012). Various molecular and microbiology based approaches have revealed the polymicrobial nature of the infections in CF with the identification of complex microbiota including bacteria, fungi, and viruses (Lynch and Bruce, 2013). Most of these microorganisms are either acquired from the environment or through contact with other infected patients (Lipuma, 2010).

Bacteria constitute the major portion of the microorganisms associated with CF. The most common bacterial inhabitants of the CF airways include *Haemophilus influenzae*, *Staphylococcus aureus*, *Pseudomonas Aeruginosa*, and *Burkholderia cepacia* complex (BCC) (Harrison, 2007; Lipuma, 2010). Among them, *P. aeruginosa* is the most dominant bacterial species known to cause chronic respiratory infections in more than 50% of adult CF patients (Coutinho et al., 2008). *P. aeruginosa* is a ubiquitous Gram-negative bacterium possessing a wide variety of pathogenicity factors to evade the host defense system (Davies, 2002). During the early stages of infection, the bacterium attaches itself to lung epithelial cell surface receptors through specific adhesins and secretes extracellular products to prolong its survival in the CF airways (Tang et al., 1995). The extracellular products secreted by *P. aeruginosa* include enzymes such as elastase and alkaline protease, exotoxins, siderophores, and phenazines such as pyocyanin with a known role in virulence (Haas et al., 1991). Moreover, *P. aeruginosa* cells form biofilms in order to proliferate inside the lungs and protect themselves from antibiotic agents (Singh et al., 2000).

In addition to bacteria, some fungal species are also known to colonize the respiratory tracts of CF patients (Cimon et al., 2000; Pihet et al., 2009). Mycological examination of the specimens obtained from CF patients have shown that *Aspergillus fumigatus* is the most predominant fungal colonizer of the CF lungs as it has been recovered from 6 to 71% of CF patients (Bakare et al., 2003; Horre et al., 2010). However, the presence of non-*Aspergillus* fungal species often remains unnoticed owing to the lack of sensitive culture techniques to examine the sputum specimens from CF patients (Delhaes et al., 2012). Recently, a more targeted approach has been developed by combining molecular techniques with laboratory culture methods, which can now identify a wide range of fungal pathogens in the expectorated sputa (Middleton et al., 2013). Studies conducted on CF patients in Australia and certain parts of Europe have confirmed the emergence of a new fungal genus *Scedosporium* (originally called *Pseudallescheria*) that causes infections in the lungs of immunocompromised hosts (Blyth et al., 2010a; Paugam et al., 2010; Lackner et al., 2014). *Scedosporium* sp. have been isolated from the sputum specimens of 14.7–17.4% of Australian CF patients which makes it the second most common fungal respiratory pathogen associated with CF (Blyth et al., 2010a,b). *Scedosporium aurantiacum* is a recently identified, highly virulent member of the *Scedosporium* sp. complex recovered from one in six CF patients in Sydney (Heath et al., 2009; Blyth et al., 2010b; Harun et al., 2010). The clinical consequences of the *S. aurantiacum* colonization or infections in the CF patients remain to be explored (Harun et al., 2010).

According to the clinical reports, the prevalence of fungi in the respiratory tracts of CF patients is mainly affected by the bacteria present, and the interactions between the bacteria and fungi potentially impact the disease outcome (Sibley et al., 2006; Chotirmall et al., 2010; Leclair and Hogan, 2010). Several *in vitro* studies have reported an inhibitory effect of *P. aeruginosa* against the common lung co-inhabitants such as *A. fumigatus* or the yeasts *Candida albicans*, and *Cryptococcus neoformans* (Hogan and Kolter, 2002; Bandara et al., 2010; Cugini et al., 2010). Similar data for *S. aurantiacum* are lacking. Reflecting the increasing importance of *S. aurantiacum* in CF, we examined the effect of clinical *P. aeruginosa* CF isolates PASS1 and PASS2 and laboratory reference strain PAO1 on the growth of two clinical *S. aurantiacum* isolates WM 06.482 and WM 08.202 using solid plate assays and liquid co-cultures containing medium that mimics the nutritional content of human CF sputum (Palmer et al., 2007).

## Materials and Methods

### Growth and Maintenance of Strains

Strains used in the study are listed in **Table 1**. *P. aeruginosa* PASS1 and PASS2 were isolated from the sputum samples of CF patients (Penesyan et al., under review). A common laboratory ‘reference’ strain PAO1 (Lewenza et al., 2014) was also included in the study. *S. aurantiacum* strains WM 06.482 and WM 08.202 were obtained from the culture collection of the Medical Mycology Research Laboratory, Centre for Infectious Diseases and Microbiology, Westmead Hospital, Sydney, NSW, Australia (Kaur et al., 2015). Virulence levels of all *P. aeruginosa* strains used in this study have been tested previously using *Caenorhabditis elegans* based infection model (Lewenza et al., 2014; Penesyan et al., under review). Virulence studies of *S. aurantiacum* have been performed using *Galleria mellonella* larvae model (Kaur et al., 2015).

**Table 1:** *Pseudomonas aeruginosa* and *Scedosporium aurantiacum* strains used in the study.

Strain	Strain name	Source	Virulence level	Reference
PASS1	*P. aeruginosa*	Sputum sample of a cystic fibrosis (CF) patient Sydney, NSW, Australia	High	Penesyan et al. (under review)
PASS2	*P. aeruginosa*	Sputum sample of a CF patient Sydney, NSW, Australia	Low	Penesyan et al. (under review)
PAO1 (ATCC 15692)	*P. aeruginosa*	Wound exudate Melbourne, VIC, Australia	High	Holloway (1955)
WM 06.482	*S. aurantiacum*	Invasive clinical isolate from CF patient Sydney, NSW, Australia	High	Kaur et al. (2015)
WM 08.202	*S. aurantiacum*	Type strain from a wound exudate Santiago de Compostela (Spain)	Low	Kaur et al. (2015)

*Pseudomonas aeruginosa* strains were revived from frozen stocks stored at -80°C by streaking on LB (Luria Bertani, Sigma) plates and incubation overnight at 37°C. Bacterial colonies were inoculated into LB broth and incubated at 37°C on an orbital shaker (200 rpm) overnight. Following fractions were prepared from overnight cultures of the *P. aeruginosa* strains: (1) Heat killed cells were obtained by incubating 1 ml of an overnight cell culture at 80°C for 60 min. Absence of any viable cells was confirmed by plating on LB agar medium; (2) Cell lysates were obtained after sonicating the cells (50 ml) on ice for 10 min in an ultrasonic processor followed by collection of the supernatant after centrifugation at 10,000 × *g* for 30 min; (3) Cell culture supernatants were collected by centrifuging 50 ml of overnight cultures of *P. aeruginosa* strains at 10,000 × *g* for 30 min. Supernatants were then freeze dried and resuspended in 100 μl of 1x PBS and stored at 4°C until use.

Fungal strains were maintained on PDA (potato dextrose agar, BD, Difco^TM^) plates at 37°C. After 5 days of growth, the conidia were scraped into sterile saline solution (0.9% w/v NaCl and 0.01% v/v Tween 80) and the suspension was filtered through a sterile cotton wool to separate the conidia from the hyphal debris. Conidia were washed with 1x PBS to remove traces of saline and the inoculum was adjusted to a McFarland standard concentration of 2.5 × 10^5^ conidia/ml. Concentration of conidia was confirmed using Neubauer counting chamber and additional plate counting.

### Construction of Strains Tagged with Fluorescent Proteins

#### *Pseudomonas aeruginosa* Strain Expressing Yellow Fluorescent Protein (YFP)

Plasmid pUCP*yfp* (Gloag et al., 2013) encoding yellow fluorescent protein (YFP) was used to transform the *P. aeruginosa* PASS1 strain. In order to make electrocompetent cells, PASS1 was cultured in 5 ml of LB broth overnight at 42°C and 200 rpm. Cells were harvested by centrifugation (14,000 *g* for 15 min at 4°C) and the ionic strength of the suspension was reduced by rigorous washing with 1x M9 minimal salts medium (Sigma) followed by two washes with ice-cold sterile milliQ water. Bacterial cells were transformed by electroporation as described by Dower et al. (1988) by adding 1 μg of the plasmid DNA to 20 μl of the washed cell aliquots. At the end of the procedure, cells were streaked on LB plates containing 8 mg/ml ampicillin and incubated for up to 48 h at 37°C to select for the transformants.

#### Construction of the *S. aurantiacum* Strain Expressing mCherry

The *mCherry* gene was PCR amplified from the pmcherry-c1 vector (Clontech Laboratories, USA) using *mCherry.fwd* and *mCherry.rev* primers (**Table 2**) and was expressed under the *Trichoderma reesei* pyruvate kinase (*pki*) promoter, which was amplified from the pCBH1corlin vector (Te’o et al., 2000) using *pki.fwd* and *pki.rev* primers. In addition, a DNA fragment featuring the *pki* promoter together with the hygromycin B resistance gene (*pki-hph*) was PCR amplified using primer *pkihph.fwd* and *pki-hph.rev* to allow selection of transformants. The fragments were engineered to contain restriction sites as needed (**Table 2**).

**Table 2:** Sequence of primers used for the construction of transformation cassettes.

Primer name	Sequence (5′–3′)
*mCherry. fwd*	**GAA GAACCT CTT AAC CTC TAG (*pki* sequence)** ATG GTG AGC AAG GGC GAG G
*mCherry. rev*	CAT GCG GGT ACC (*Kpn***I**) CTA TTA CTT GTA CAG CTC GTC CAT GC
*pki. fwd*	TGC TGC GAT ATC (*Eco*R**V**) CTT AAG TTA G TA ACT AGT GGA TC
*pki.rev*	**CTC GCC CTT GCT CAC CAT (*mCherry* sequence)** CTA GAG GTT AAG AGG TTC TTC
*pki-hph. fwd*	TAC GCG GCG CGC CCT TAA G **(***Afl***II)** TT AG T AAC TAG TGG ATC
*pki-hph.rev*	CAT GCT AAG CTT **(***Hind***III)** CTA TTC CTT TGC CCG CGG AC

*The primers contain engineered restriction sites shown in shading and the overlapping sequences are shown in bold*.

The primers *pki.fwd* and *mCherry.rev* were used to fuse the separately amplified *pki* and *mCherry* fragments in an overlap extension PCR as described by Thornton (2015). The fragment *pki-hph* was digested with restriction enzymes *Hind*III and *Afl*II (Fermentas, Thermo Scientific, USA) and fragment *pki-mcherry* was digested with *EcoR*V and *Kpn*I. The digested products *pki-hph* and *pkimcherry* were gel purified using QIAquick gel extraction kit (Qiagen, USA) and inserted into MCS-1 (multiple cloning site) and MCS-2 of the pETDuet-1 plasmid, respectively (Supplementary Figure S1). Finally, the purified vectors and inserts were ligated using T4 ligase (Fermentas, USA) at a 1:3 molar ratio for 2 h at room temperature. The final ligated vector (pETDuet-phpm) was introduced into *Escherichia coli* DH5α competent cells as described by Inoue et al. (1990). Selection of transformants was performed on LB agar plates containing ampicillin (100 μg/ml) and incubating at 37°C. Selected transformants were grown in 3 ml of LB and plasmid DNA was isolated using QIAprep Spin Miniprep kit (Qiagen, USA). The plasmid pETDuet-phpm was sequenced by AGRF, Sydney, NSW, USA to check sequence alignment of the inserted gene cassettes.

The pETDuet-phpm DNA was introduced into highly virulent *S. aurantiacum* WM 06.482 using protoplast-mediated transformation based on the method adopted from Penttilä et al. (1987) with modifications. The young hyphae obtained from an overnight culture of WM 06.482 on PDA plates with cellophane at 28°C were digested with 10 mg/ml of lysing enzyme from *T. harzianum* (Sigma–Aldrich, Australia) to obtain protoplasts which were then filtered through a sterile sintered glass filter (porosity 1). Osmotically stabilized protoplasts were transformed with 5 μg of plasmid DNA as described by Penttilä et al. (1987). Transformed protoplasts were mixed with 10 ml of molten agar (1.5% w/v KH_2_PO_4_, 0.5% w/v NH_4_SO_4_, 2% w/v glucose, 1 M sorbitol, pH 5.5) containing hygromycin B (410 U/ml) and overlayed onto PDA plates which were incubated at 28°C for 3–5 days. Hygromycin resistant colonies were restreaked onto fresh PDA plates containing hygromycin B (410 U/ml) for a second round of selection. Transformation efficiency was calculated as number of transformants per μg of plasmid DNA. Expression of the mCherry protein in selected transformants was confirmed using Fluoview FV1000 inverted confocal microscope (Olympus) with an excitation and emission wavelength 488/633 nm (HeNe).

### Growth Inhibition Assays

The effect of *P. aeruginosa* on the growth *of S. aurantiacum* was tested in different combinations on both solid and liquid growth media. Combinations of bacterial and fungal strains for the testing are presented in **Table 3**.

**Table 3:** Types of cultures used to investigate the effect of different *P. aeruginosa* strains on *S. aurantiacum*.

Type of co-culture	*P. aeruginosa* strains	*S. aurantiacum* strains
Solid plate (cross streak, disk inhibition assay)	PASS1, PASS2, PAO1	WM 06.482, WM 08.202
Liquid cultures (chemical fluorescent dyes)	PASS1, PASS2, PAO1 (stained with Syto9)	WM 06.482, WM 08.202 (stained with Mito tracker FR)
Liquid culture (genetically tagged strains)	PASS1 (*yfp*-labeled)	WM 06.482 *(mCherry-* labeled)
Liquid culture (addition of an antibiotic)	PASS1 (*yfp*-labeled)	WM 06.482 *(mCherry-* labeled)

#### Cross Streak Assay using Live Cells

The effect of bacteria on fungal growth was assessed using an agar plate method described by Kerr (1999), with slight modifications adopted from Chen et al. (2013). *P. aeruginosa strains* PASS1, PASS2, and PAO1; and *S. aurantiacum* strains WM 06.482 and WM 08.202, were cultured together on a synthetic cystic fibrosis medium (SCFM) that mimics the nutritional content of human CF sputum. SCFM contains average concentrations of ions, free amino acids, glucose, and lactate present in the CF sputum samples (Palmer et al., 2007). Solid SCFM agar plates were made with an addition of 2% w/v agar to liquid SCFM medium. A sterile cotton swab was used to draw a straight vertical line of *P. aeruginosa* cells (1 × 10^8^ CFU/ml=0.5 McFarland standard concentration) across the plate. At the same time, *S. aurantiacum* conidia (2.5 × 10^5^ conidia/ml = 0.5 McFarland standard concentration) were inoculated with a cotton swab horizontally across the upper part of the plate preventing any direct contact between fungi and bacteria. The plates were dried at room temperature for 15 min and incubated at 37°C. Digital photography was performed after 24 h to visualize the growth of both bacterial and the fungal strains tested on the plate.

#### Disk Inhibition Method using Live Cells and Cell Fractions

Sterile filter paper disks (Whatman no. 1; Sigma–Aldrich), 7 mm in diameter, were impregnated with 20 μl of the *P. aeruginosa* PASS1, PASS2, and PAO1 cell fractions, i.e., cell lysates, cell culture supernatant and heat inactivated cells (see preparation in section 1.1) and placed on an SCFM plate that was freshly surface seeded with 100 μl (2.5 × 10^5^ conidia/ml) of *S. aurantiacum* conidia (WM 06.482 or WM 08.202). A suspension of live *P. aeruginosa* cells was included for comparison. The plates were incubated at 37°C for up to 3 days and observed at regular intervals for the appearance of any clear inhibition zones around the disks. Assays were repeated in three biological replicates. A relative inhibition index was calculated for each *P. aeruginosa* isolate by dividing the area of activity (difference between the area of the inhibition zone and area of the colony) by the area of the colony.

#### Effect of Bacteria on the Fungal Growth in Liquid Co-cultures

Interactions between *P. aeruginosa* and *S. aurantiacum* were observed in liquid medium using both chemical fluorescent stains and genetically labeled strains of bacteria and fungi in a direct contact with each other. In case of fluorescently labeled co-cultures, 1 × 10^8^ CFU/ml of *P. aeruginosa* PASS1, PASS2, and PAO1 and 2.5 × 10^5^ conidia/ml of *S. aurantiacum* WM 06.482 and WM 08.202 were inoculated in 20 ml SCFM medium in 100 ml shake flasks and incubated for 24 h at 37°C on an orbital shaker at 150 rpm. Aliquots were taken on a sterile glass slide from the co-cultures after every 4 h, washed with 1x PBS and fixed using 2% v/v paraformaldehyde (Sigma–Aldrich). The co-cultures were stained with DNA specific Syto9 (0.6 μM) and mitochondria specific Mito-Tracker^R^ Red FM (25 nM) for 15 min in the dark as per the manufacturer’s protocol (Molecular Probes, Life Technologies). Bacterial cells were expected to stain with Syto9 whereas fungal cells would stain with Mito-Tracker^R^ Red FM. Fixed specimens were imaged using Fluoview FV1000 inverted confocal microscope (Olympus) with an excitation and emission wavelength of 488 nm (Ar) and 633 nm (HeNe).

The genetically tagged *P. aeruginosa* PASS1yfp strain and *S. aurantiacum* WM 06.482mCherry strain were also cultured together in 20 ml of SCFM for 24 h at 37°C, shaking at 150 rpm. At the end of the incubation period, cells were washed and fixed on sterile glass slides as above. Imaging was performed with a confocal microscope using an excitation and emission wavelength of 488 nm (blue laser diode for yfp) and 561 nm (yellow–green laser for mCherry) respectively. Liquid co-cultures with genetically labeled PASS1 and WM 06.482 strains were also repeated by adding different concentrations of gentamicin (2.5–10 mg/ml), which is a commonly used antibiotic against bacteria (Doring et al., 2000; Lin et al., 2011). Image analysis for both types of co-cultures was performed using IMARIS imaging software.

#### Transwell Assay with Polycarbonate Membranes

In order to explore the role of secreted bacterial metabolites on the fungi, *P. aeruginosa* strains PASS1, PASS2, and PAO1 and *S. aurantiacum* strains WM 06.482 and WM 08.202 were co-cultured in SCFM in sterile six-well Transwell plates (Corning) with polycarbonate cell culture inserts (0.4 μm, Sigma–Aldrich) in order to prevent direct contact between the fungal and bacterial strains. *P. aeruginosa* (1 × 10^8^ CFU/ml) and *S. aurantiacum* (2.5 × 10^5^ conidia/ml) were inoculated in the bottom and top of the membrane insert, respectively. The plates were incubated at 37°C for 24 h and any inhibition of the growth of *S. aurantiacum* was measured as a difference in the dry weight of the *S. aurantiacum* cultured with or without *P. aeruginosa*. The method of calculating dry weight was adopted from Kaur et al. (2015).

### Effect of Phenazines

Phenazines were extracted from all *P. aeruginosa* strains (PASS1, PASS2, and PAO1) cultured in 5 ml of LB (in three biological replicates) for 2 days at 37°C using chloroform according to a method described by Mavrodi et al. (2001). Crude phenazine extracts were dried under reduced pressure to remove the solvent, resuspended in 80% acetonitrile (ACN) and applied to the filter paper disks (Whatman paper no. 1). The presence of pyocyanin in the crude phenazine extracts was confirmed using Ultra High Performance Liquid Chromatography (UHPLC) as described by Penesyan et al. (under review). While the absolute concentration of pyocyanin in the crude phenazine extracts from the three *P. aeruginosa* strains was not known, major experimental discrepancy was minimized by using same amount (20 μl) of crude extracts for the testing. The effect of these crude extracts on the fungal growth was determined using a disk inhibition assay (as described in section Disk Inhibition Method using Live Cells and Cell Fractions) where disks containing 20 μl of phenazine extracts from different *P. aeruginosa* isolates were air-dried and placed on SCFM agar plates freshly spread with 2.5 × 10^5^ conidia/ml of *S. aurantiacum* strains (WM 06.482 and WM 08.202). The activity of blank 80% ACN, blank LB medium extract and the solution of commercial pyocyanin (10 mM, Sigma–Aldrich) were also tested against *S. aurantiacum* for comparison. Plates were incubated for 48 h at 37°C and observed for the presence of clearing zones around the filter paper disks as an indication of inhibitory activity of the extracts on fungal growth.

### Statistical Analysis

Statistical significance between the means of different experimental datasets was analyzed using two-tailed Student’s *t*-test. SD with *p*-value less than 0.05 was considered significant. All experiments were performed in biological triplicates.

## Results

### Inhibition of *S. aurantiacum* Growth by Live *P. aeruginosa* Cells

**FIGURE 1:**
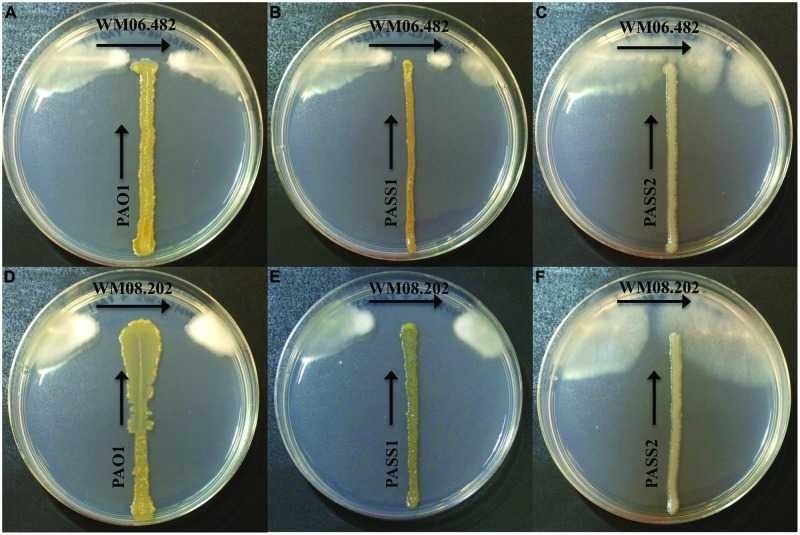
**Cross-streak plate assay between different strains of *Pseudomonas aeruginosa* and *Scedosporium aurantiacum* on synthetic cystic fibrosis medium (SCFM) agar plates**. All bacterial strains were inoculated vertically whereas the fungal strains were streaked horizontally across the upper part of the SCFM agar plate. The plates were incubated at 37°C for 24–48 h. **(A–C)** Inhibition of *S. aurantiacum* strain WM 06.482 by PAO1, PASS1, and PASS2 strains of *P. aeruginosa*. **(D–F)** Inhibition of *S. aurantiacum* strain WM 08.202 by PAO1, PASS1, and PASS2 strains of *P. aeruginosa*.

When *S. aurantiacum* strains WM 06.482 (high virulence) and WM 08.202 (low virulence) were cross streaked against three different *P. aeruginosa* isolates PAO1, PASS1, and PASS2 on the SCFM agar medium, an area of inhibition was observed in the growth of both *S. aurantiacum* strains after 24 h (**Figures 1A–F**). It was evident from the size of the inhibition area that the bacterial strains had lesser impact on the highly virulent *S. aurantiacum* strain WM 06.482 (**Figures 1A,B**) compared to the less virulent WM 08.202 strain (**Figures 1D,E**). Out of the three bacterial strains studied, *P. aeruginosa* PASS2 had the weakest inhibitory effect on the *S. aurantiacum* strains in the plate test as seen in **Figures 1C,F**.

### Effect of *P. aeruginosa* Cell Extracts on *S. aurantiacum*

**FIGURE 2:**
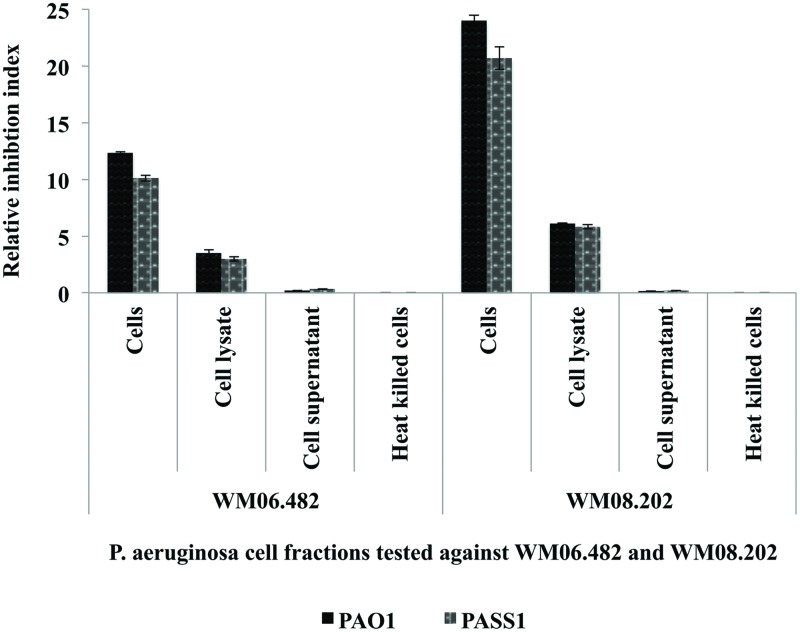
**Susceptibility of *S. aurantiacum* (WM 06.482 and WM 08.202) to *P. aeruginosa* (PAO1, PASS1, and PASS2) and their cell lysate fractions**. Relative inhibition index was calculated as the average value of three replicates (*n* = 3) with a *p*-value <0.05 considered as significant.

The effect of different cell fractions, i.e., the culture supernatant and cell lysate, and heat inactivated cells of *P. aeruginosa* strains PASS1, PASS2, and PAO1 was further tested on the growth of *S. aurantiacum* WM 06.482 and WM 08.202 using the disk inhibition method. Following 48 h incubation, clear inhibition zones were observed on plates inoculated with living cells of *P. aeruginosa* PASS1 and the reference strain PAO1 and their respective cell lysates. The inhibitory effect of *P. aeruginosa* was expressed as a relative inhibition index (**Figure 2**).

Living cells of both PAO1 and PASS1 and their corresponding cell lysates displayed a higher inhibitory activity against the less virulent *S. aurantiacum* strain WM 08.202 compared to the high virulence strain WM 06.482. Cell supernatants and heat killed *P. aeruginosa* cells failed to elicit a response against either of the fungal strains. In a separate experiment, the effect of *S. aurantiacum* was also tested against *P. aeruginosa* by incubating filter disks impregnated with *S. aurantiacum* conidia and cell fractions on the plates freshly seeded with *P. aeruginosa* cells. As *S. aurantiacum* failed to display any inhibition against *P. aeruginosa*, these interactions were not studied further (data not shown).

### Effect of *P. aeruginosa* on Fungal Physiology

**FIGURE 3:**
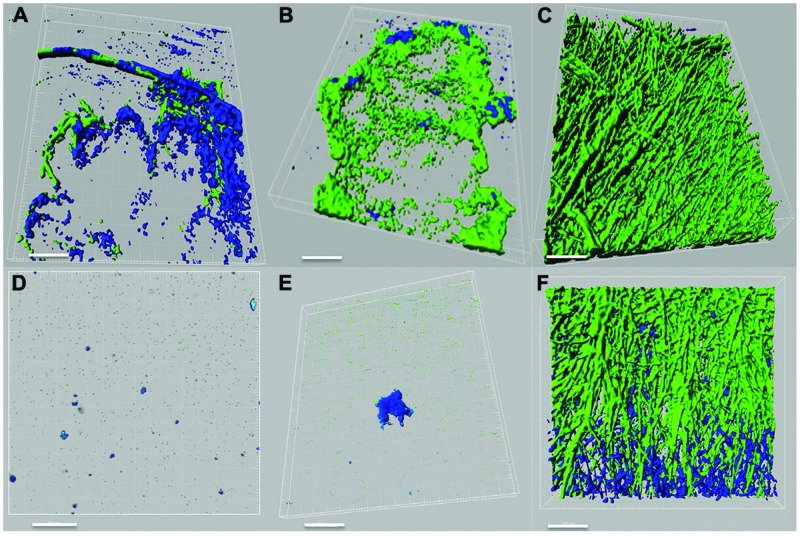
**Confocal laser scanning microscope (CLSM) images of interactions between *P. aeruginosa* (PAO1, PASS1, and PASS2) and *S. aurantiacum* (WM 06.482 and WM 08.202) as observed after co-incubating both the organisms in SCFM liquid medium at 37°C for 24 h**. *P. aeruginosa* cells are stained with Syto9 (shown in green) and *S. aurantiacum* with Mito-tracker deep red FM (shown in blue). 3D re-construction of CLSM datasets was performed using IMARIS software package (Bitplane). Scale bar = 50 μm. **(A–C)** CLSM images of co-culture of WM 06.482 with PAO1, PASS1, and PASS2, respectively. **(D–F)** CLSM images of co-culture of WM 08.202 with PAO1, PASS1, and PASS2, respectively.

*Pseudomonas*–*Scedosporium* interactions were also studied using confocal microscopy by imaging cellular aggregates from liquid co-cultures labeled with fluorescent stains. Confocal images demonstrated an inhibitory effect of the *P. aeruginosa* PASS1 (isolated from sputum of a CF patient) and the reference strain PAO1 on the growth and development of both *S. aurantiacum* strains tested (**Figures 3A–F**). In the course of 24 h, the bacteria had attached to the surface of fungal hyphae and formed biofilm-like structures containing a high density of bacterial cells but very few fungal hyphae. The tested bacterial strains had a weaker impact on the more virulent WM 06.482 compared to the less virulent WM 08.202, showing the resistant nature of the more virulent strain also highlighted in the plate tests. Although different fluorescent stains were used to distinguish between *P. aeruginosa* and *S. aurantiacum* in liquid cultures, it was difficult to visualize the detailed effect of bacteria on the fungal hyphae due to permeabilisation of the Syto9 dye by both *P. aeruginosa* and *S. aurantiacum*. No growth inhibiting effect was observed when the fungal strains were co-cultured with PASS2 as indicated by dense growth of fungi in **Figures 3C,F**. These observations were also consistent with the results seen in the assays carried on plates.

### Interactions between Genetically Tagged *P. aeruginosa* and *S. aurantiacum* Strains

**FIGURE 4:**
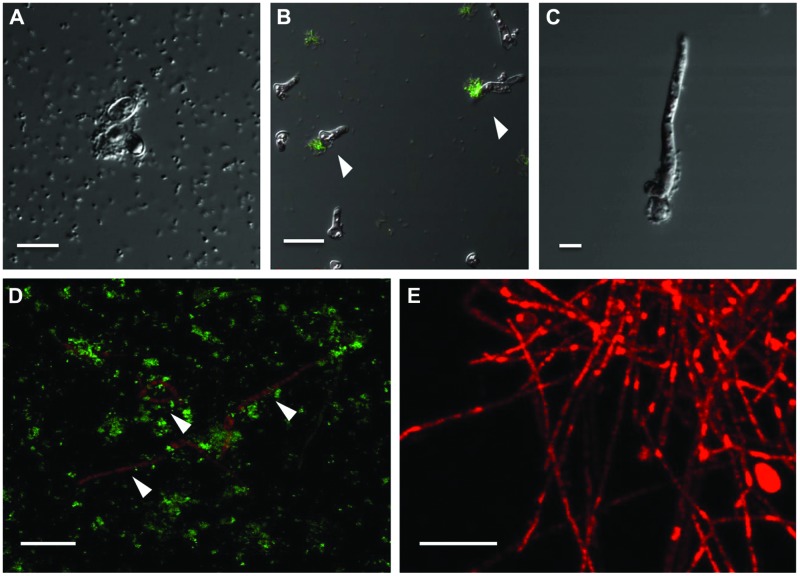
**Adhesion and colonization of mCherry-tagged *S. aurantiacum* strain WM 06.482 (shown in red) by *P. aeruginosa* strain PASS1 tagged with yfp (shown in green) during coculturing in SCFM for 24 h at 37°C**. Scale bar = 20 μm. **(A)** *P. aeruginosa* cells adhered to germinating *S. aurantiacum* conidia after 2 h of incubation as viewed by DIC. **(B,C)** after 8 h, some young hyphae were surrounded by bacterial cells. **(D)** Bacteria can be seen attached to the hyphal filaments after incubation for 24 h. **(E)** Healthy growing culture of WM 06.482 expressing *mCherry* in the absence of bacteria. ^∗^White arrows indicate fungal filaments that are being colonized by the bacteria.

To circumvent the difficulty in differentiating between bacteria and fungi in liquid co-cultures, genetically tagged *P. aeruginosa* strain PASS1 expressing yfp and *S. aurantiacum* strain WM 06.482 expressing mCherry were developed. With this arrangement, it was observed that the bacteria started colonizing the fungal conidia soon after incubating them together in the SCFM (**Figure 4A**). Thus, within 8 h, bacteria began aligning themselves along the length of fungal hyphae as seen in **Figures 4B,C**. After 24 h, large clumps of *P. aeruginosa* cells were observed on *S. aurantiacum* hyphal filaments (**Figure 4D**), and the amount of hyphae was also reduced in number compared to the *S. aurantiacum* control without the bacteria (**Figure 4E**).

### Effect of Antibiotics used in Clinical Practice on Co-cultures

**FIGURE 5:**
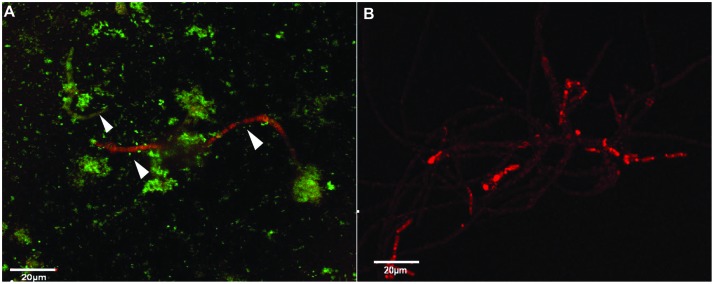
**The effect of gentamicin on *P. aeruginosa* (PASS01) and *S. aurantiacum* (WM 06.482) co-cultures growing in SCFM at 37°C for 24 h**. Scale = 20 μm. **(A)** Co-culture of WM 06.482 and PASS01 without the antibiotic. **(B)** Active growth of *S. aurantiacum* in a co-culture treated with 8 mg/ml of gentamicin to eradicate the bacterial growth.

Analysis of the plate cultures and confocal images confirmed that *P. aeruginosa* had an inhibitory effect on the growth of *S. aurantiacum*. Therefore, in order to further validate this finding and to reveal the possible effect of antibiotic therapy on *S. aurantiacum* and *P. aeruginosa* mixed populations present in CF lungs, co-culturing was repeated with an addition of varying amounts of gentamicin (2.5–10 mg/ml) to selectively inhibit the growth of *P. aeruginosa S. aurantiacum-P. aeruginosa* co-cultures were also maintained without the addition of gentamicin for comparison (**Figure 5A**). All bacteria were killed at a concentration of 8 mg/ml of gentamicin. As seen from **Figure 5B**, *S. aurantiacum* strain WM 06.482 was growing actively in the absence of *P. aeruginosa* strain PASS1 indicating the reversal of the inhibitory effect caused by live bacteria against the fungus.

### Indirect (non-physical) Interactions between *P. aeruginosa* and *S. aurantiacum*

**FIGURE 6:**
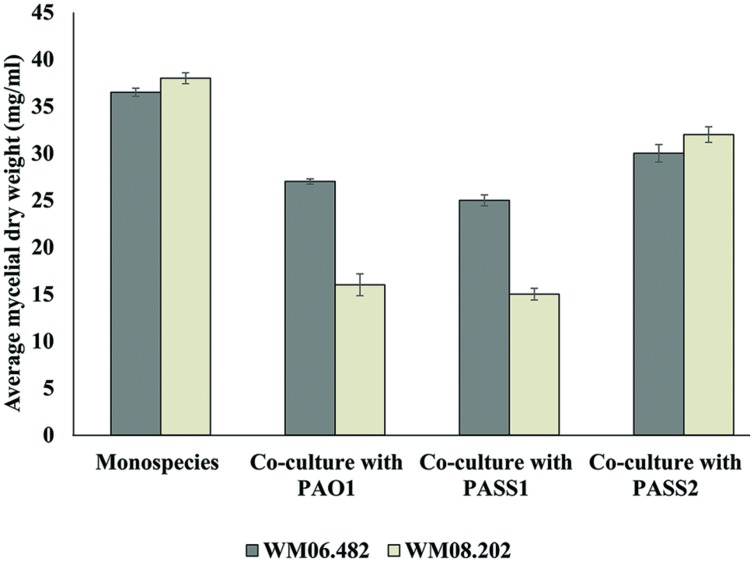
**The inhibitory effect of *P. aeruginosa* on *S. aurantiacum* during co-culture in SCFM, determined by the change in dry weight of *S. aurantiacum* hyphae**. A sterile polycarbonate membrane was used to separate *P. aeruginosa* from *S. aurantiacum* in six well culture plates. Error bars on each data point represent SEM of three independent experiments. *P*-values were calculated by student’s *t*-test, where *p* < 0.05 was considered significant.

To investigate whether physical contact between *P. aeruginosa* and *S. aurantiacum* was important to trigger growth inhibition, co-cultures were performed in six-well plates fitted with polycarbonate membranes to prevent direct contact between *P. aeruginosa* and *S. aurantiacum* cells while allowing free exchange of nutrients and extracellular molecules between the organisms. Growth of the less virulent *S. aurantiacum* strain WM 08.202 was inhibited when co-cultured with PAO1 and PASS1, evident from the substantial decrease in the fungal biomass (**Figure 6**) when compared to the culture of WM 08.202 maintained for the same amount of time.

*Pseudomonas aeruginosa* isolate PASS1 and the reference strain PAO1 showed a milder inhibitory effect against the high virulence *S. aurantiacum* strain WM 06.482. The PASS2 strain had little or almost no effect on growth of either of the *S. aurantiacum* strains. The results suggested that cell–cell contact was in fact not necessary to bring about inhibition of the growth of *S. aurantiacum* by *P. aeruginosa* and that the inhibition might involve bacterial metabolites and/or extracellular signaling molecules. In addition, *S. aurantiacum* strains WM 06.482 and WM 08.202 produced a red colored pigment when co-cultured with clinical *P. aeruginosa* strain PASS1 and reference strain PAO1. No such pigment was observed in the co-cultures involving *S. aurantiacum* and PASS2 strain (Supplementary Figure S2).

### Effect of Phenazines on the Growth of *S. aurantiacum*

**FIGURE 7:**
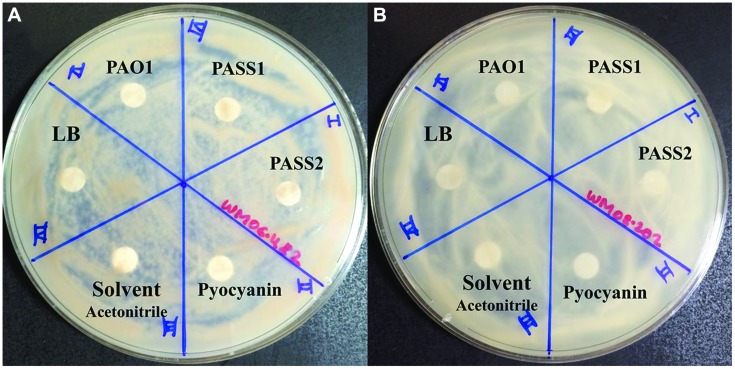
**Effect of *P. aeruginosa* phenazines on two *S. aurantiacum* isolates **(A)** WM 06.482 and **(B)** WM 08.202**. Phenazines were extracted from different *P. aeruginosa* strains (PAO1, PASS1, and PASS2) and redissolved in acetonitrile (ACN). LB medium extract, ACN solvent and commercial phenazine (pyocyanin) were also tested against *S. aurantiacum*.

To test whether known virulence factors such as phenazines secreted by *P. aeruginosa* were involved in the inhibition of *S. aurantiacum* growth, the effect of crude phenazine extracts from *P. aeruginosa* strains PAO1, PASS1, and PASS2 were tested on the two *S. aurantiacum* strains using a disk inhibition assay. No inhibition was observed with disks saturated with the crude extracts as seen in **Figure 7**. All *S. aurantiacum* strains also showed resistance to a high concentration (10 mM) of commercial phenazine pyocyanin. Phenazines are known to have an inhibitory effect against a wide range of fungal species (Kerr et al., 1999).

## Discussion

Most of the studies targeting bacterial-fungal interactions *in vitro* have been performed with bacterial laboratory reference strains using either fungus-specific culture media (PDA, SABD) and/or minimal salts medium (Kerr et al., 1999; Hogan and Kolter, 2002; McAlester et al., 2008; Bandara et al., 2010; Manavathu et al., 2014). Differently to previous studies and to provide a better focus, we used a CF sputum-mimicking medium, i.e., SCFM to explore the possible effect of *P. aeruginosa* on *S. aurantiacum* in the CF lung environment. We also used two recently isolated clinical CF strains of *P. aeruginosa*, PASS1 and PASS2, together with PAO1, a commonly used reference strain, and a clinical *S. aurantiacum* isolate (WM 06.482) with a high established virulence and a less-virulent type strain WM 08.202 to add to the clinical relevance of the findings.

Our results demonstrated that *P. aeruginosa* strains exhibit an inhibitory effect against *S. aurantiacum.* Consistent with the co-culture studies involving *P. aeruginosa* and other fungi, initial screening using plate assays suggested that presence of metabolically active (live) bacteria was necessary to inhibit the growth of the fungus as heat killed cells had no effect on *S. aurantiacum* growth (Mowat et al., 2010). Further on, extracts obtained from the bacterial monocultures failed to show any inhibitory effect. Thus it is possible that inhibition pathways might involve genes that are expressed only in bacterial-fungal co-cultures. In this respect our findings are similar to those of Rella et al. (2012) who showed that the growth of *C. neoformans* was not affected by the cell extracts obtained from *P. aeruginosa* strains PAO1 and PA14 cultured separately. The inhibition of *S. aurantiacum* by cell lysates of *P. aeruginosa* may be explained by the presence of bacterial exotoxins that are released during the cell lysis.

Confocal microscopy has been used to study interactions between chemically stained *P. aeruginosa* and major fungal lung pathogens such as *C. albicans* and *A. fumigatus* in liquid co-cultures (Bandara et al., 2010; Manavathu et al., 2014). However, the use of chemical stains was limited by the cross staining of bacteria and fungi thereby making it impossible to differentiate between them under a confocal microscope (Bandara et al., 2010). One of the key features of the current study is the use of *P. aeruginosa* and *S. aurantiacum* strains that were genetically tagged with fluorescent proteins in order to characterize the interactions in detail. To the best of our knowledge, this is the first report on successful genetic transformation of the newly described *S. aurantiacum* species. As no homologous promoters are available for this fungal species as yet, the fluorescent marker mCherry and the *E. coli hph* gene encoding hygromycin B phosphotransferase were expressed under a heterologous *pki* (pyruvate kinase) promoter derived from another ascomycetous fungus, *T. reesei* (Te’o et al., 2002; Boon et al., 2008; Klix et al., 2010). In previous studies, heterologous promoters such as *pki* and *gpdA* have been successfully used for gene expression across various phylogenetically close species (Punt et al., 1990; Jieh-Juen Yu, 1998; Ruiz-Diez and Martinez-Suarez, 1999; Almeida et al., 2007). The amount of hygromycin B required to inhibit the growth of *S. aurantiacum* was relatively high (410 U/ml) compared to some other fungi, which shows the highly resistant nature of *S. aurantiacum* also observed in antifungal susceptibility tests described in other studies (Lackner et al., 2012). Although the transformation efficiency was low (2.2 μg of plasmid DNA), transformant strains expressing the mCherry protein were obtained.

Confocal microscopy of the bacterial-fungal co-cultures revealed that bacteria elicit a specific inhibitory response by establishing a physical contact with the fungal hyphae. Similar types of interactions have also been observed in yeasts such as *C. albicans* and ascomycetous fungi such as *A. nidulans* and *Alternaria alternata* (Hogan and Kolter, 2002; Jarosz et al., 2011). This association might be directed toward utilization of the fungus by bacteria as an additional source of nutrients, or as an additional matrix support to form biofilms (Hibbing et al., 2010), or it may be a strategy to promote their own survival by inhibiting the fungal growth owing to nutrient limiting conditions in the medium (Brand et al., 2008).

Under nutrient limiting conditions, biofilm formation has been described as an important characteristic for *P. aeruginosa* mediated killing of other fungi such as *C. albicans* and *A. fumigatus* (Hogan and Kolter, 2002; Manavathu et al., 2014). Similarly, an inhibitory effect was also displayed by the biofilm forming strains of *P. aeruginosa* (PASS1 and PAO1) against the two *S. aurantiacum* strains in this study. PASS1 and PAO1 are high virulence strains, which share many similarities in their respective genomes. In contrast, the least virulent bacterial strain PASS2 (Penesyan et al., under review) that failed to show an effect against the fungi lacks several virulence related genes such as those encoding phenazines and the *psl* (polysaccharide synthesis locus) gene cluster which is required for biofilm formation (Ma et al., 2009; Penesyan et al., under review). The effect of bacteria on the growth of the less virulent *S. aurantiacum* strain WM 08.202 was much higher compared to the more virulent WM 06.482 both in the plate assays and in liquid co-cultures. This difference probably results from their different physiology as shown by Kaur et al. (2015) and possibly higher resistance to antifungals of the more virulent *S. aurantiacum* strain WM 06.482. These factors will be studied further when annotated *S. aurantiacum* genomes are available.

While biofilm formation and colonization of fungal hyphae in the nutrient limited SCFM liquid medium clearly contributed to the inhibition of *S. aurantiacum* by *P. aeruginosa*, it was not absolutely essential for the inhibitory effect as the cross streak assay with cultures not touching each other and disk inhibition experiments using cell lysates also resulted in inhibition of fungal growth. These indicated the possible involvement of secreted diffusible bacterial exoproducts/metabolites in fungal growth inhibition. One of these metabolites pyocyanin, a phenazine, is an extracellular redox-active virulence factor which is widely known to affect the growth of a large number of fungal species such as *A. fumigatus*, *C. albicans*, and *C. neoformans* (Kerr et al., 1999; Laursen and Nielsen, 2004; Gibson et al., 2009). Corroborating the highly resistant nature of *S. aurantiacum*, the amount of commercial pyocyanin (i.e., 10 mM) included in the test for comparison, was much higher than the MIC (minimum inhibitory concentration) of pyocyanin used for *C. albicans* and *A. fumigatus* (>0.3 mM; Kerr et al., 1999). These amounts are significantly higher than the amount of pyocyanin normally detected in the lungs of CF patients (100 μM; Wilson et al., 1988). However, neither crude phenazines nor pyocyanin showed an inhibitory effect against *S. aurantiacum* in our assays. A similar phenomenon has been observed in some ascomycetous fungi such as *A. sclerotiorum* (Hill and Johnson, 1969). Although it is not yet known if the phenazines are modified or sequestered by *S. aurantiacum*, the production of a red colored pigment in co-cultures could be due to a detoxification mechanism used by the fungus against bacterial phenazines. However, further studies into the chemical structure and UV and visible absorption spectra are required in order to ascertain if the red pigment indicates a modified phenazine.

In addition to phenazines*, P. aeruginosa* has also been reported to produce a wide variety of other exoproducts/metabolites such as proteases, elastases, haemolysin, and rhamnolipids that contribute to bacterial virulence (McAlester et al., 2008; Ben Haj Khalifa et al., 2011; Heeb et al., 2011; Rella et al., 2012; Mear et al., 2013). Their possible activity against *S. aurantiacum* will be worthy of a further study.

Most of the CF associated filamentous fungal species have been isolated from the lungs of patients with prolonged antibiotic therapies (Bakare et al., 2003). Previous clinical reports by Blyth et al. (2010b) have also showed an increased prevalence of *S. aurantiacum* in CF patients administered with antibacterial drugs indicating that the presence of bacteria has an effect on the susceptibility of the lungs to fungal infection. In support of this view, an increase in the growth of the fungus was observed upon a decline in the bacterial growth through addition of gentamicin to the co-culture medium in the present study. Therefore, it seems that the *P. aeruginosa* strains prevalent in CF patients during early stages of CF hinder fungal infection of lungs by inhibiting their growth.

## Conclusion

We have assessed the effect of clinically relevant strains of *P. aeruginosa* on a newly discovered fungal lung pathogen *S. aurantiacum* in a synthetic lung-mimicking medium (SCFM) that closely resembles the chemistry of CF sputum. An inhibitory effect of *P. aeruginosa* was observed on the growth of *S. aurantiacum*, which can be mediated by the production of biologically active metabolites. Biofilm formation and colonization of fungal hyphae by bacteria were also important for *S. aurantiacum* growth inhibition. Surprisingly, the toxic *P. aeruginosa* phenazine pigments, such as pyocyanin, known to have an inhibitory effect against other fungal species including *A. fumigatus* and *C. albicans*, proved to be ineffective against *S. aurantiacum*. This suggests involvement of other virulence determinants and emphasizes the resilient nature of *S. aurantiacum* compared to other fungi present in lung infections. Further research may include transcriptomic studies of *P. aeruginosa* – *S. aurantiacum* co-cultures in order to reveal detailed molecular mechanisms underlying these interactions; these studies will be facilitated by the upcoming annotated *S. aurantiacum* genome.

## Author Contributions

Conceived and designed the experiments: JK, LK, AP, AS, IP, HN. Performed the experiments: JK, SK, BP, MK. Analysed the data: JK, AP, HN. Wrote the paper: JK, HN.

## Conflict of Interest Statement

The authors declare that the research was conducted in the absence of any commercial or financial relationships that could be construed as a potential conflict of interest.
