# Supplementary material for: Pseudomonas aeruginosa inhibits the growth of Scedosporium aurantiacum, an opportunistic fungal pathogen isolated from the lungs of cystic fibrosis patients
Source: Front Microbiol. 2015 Aug 24;6:866. doi: 10.3389/fmicb.2015.00866 (PMC4547459; doi:10.3389/fmicb.2015.00866)
Supplement: Supplementary file 1 [file Data_Sheet_1.DOCX]

***Supplementary Material***

***Pseudomonas aeruginosa* inhibits the growth of *Scedosporium aurantiacum*, an opportunistic fungal pathogen isolated from the lungs of cystic fibrosis patients**

Jashanpreet Kaur^1,2^, Bhavin Pethani^1,2^, Sheemal Kumar^1,2^, Minkyoung Kim^1,2^, Anwar Sunna^1,2^, Liisa Kautto^1,2^, Anahit Penesyan^1,2^, Ian T. Paulsen^1,2^, Helena Nevalainen^1,2^*

^1^Department of Chemistry and Biomolecular Sciences, Macquarie University, Sydney, Australia

^2^Biomolecular Frontiers Research Centre, Macquarie University, Australia

***Correspondence:**

Prof. Helena Nevalainen
Department of Chemistry and Biomolecular Sciences

Macquarie University

North Ryde, Sydney, 2109

Australia

[helena.nevalainen@mq.edu.au](mailto:helena.nevalainen@mq.edu.au)

## Supplementary Figure S1


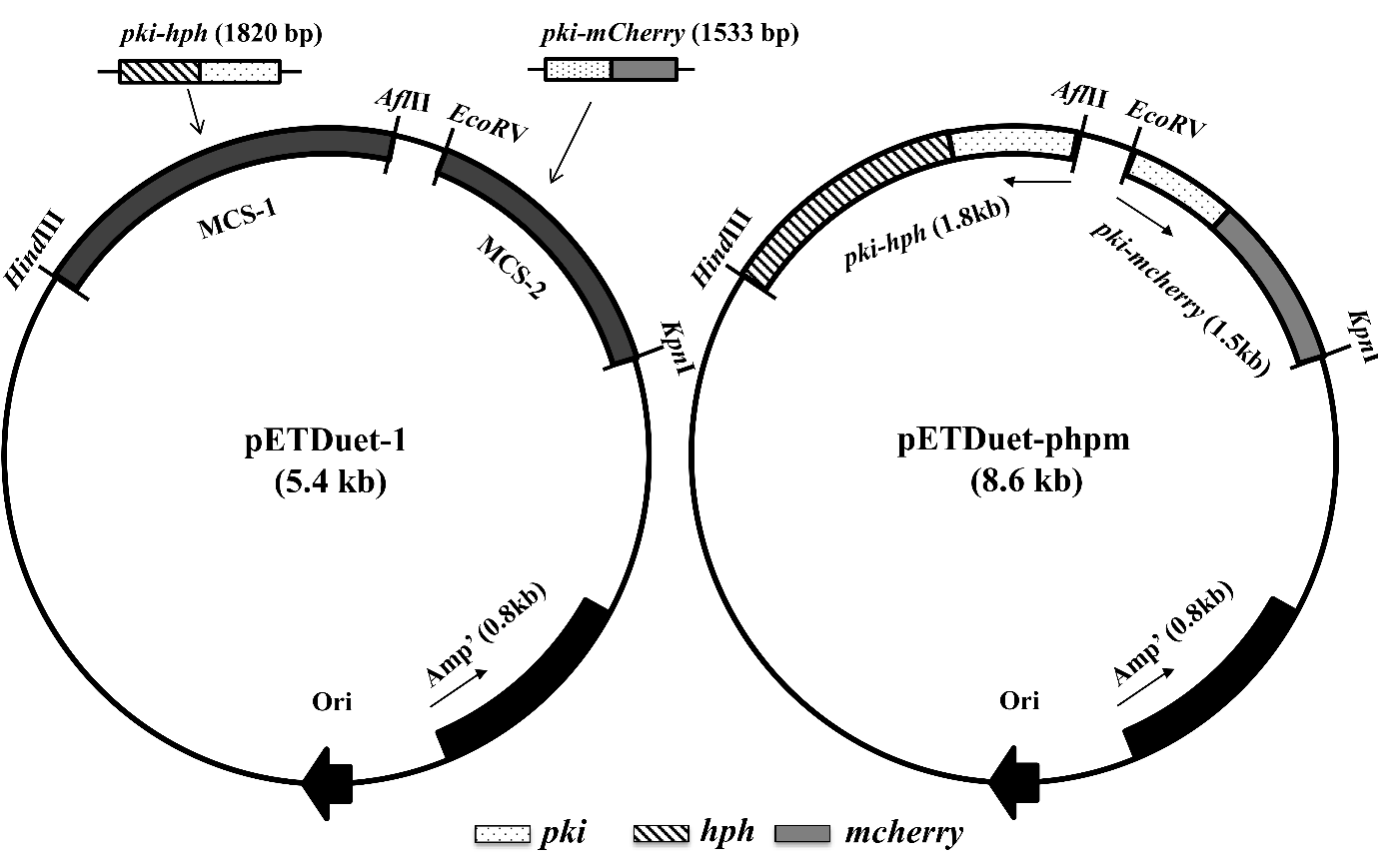


**Supplementary Figure 1.** **Schematic representation of the construction of plasmid pETDuet-phpm containing *pki-hph* (1.8 kb) with *Hind*III and *Afl*II restriction sites and *pki-mcherry* (1.5 kb) with *Eco*RV and *Kpn*I restriction sites.** The plasmid is 8.6 kb long and was made by insertion of *pki-hph* and *pki-mcherry* fragments in two different multiple cloning sites of the pETDuet-1 vector. The patterns used in the image are described in the key below.

1. **Supplementary Figure S2**

**
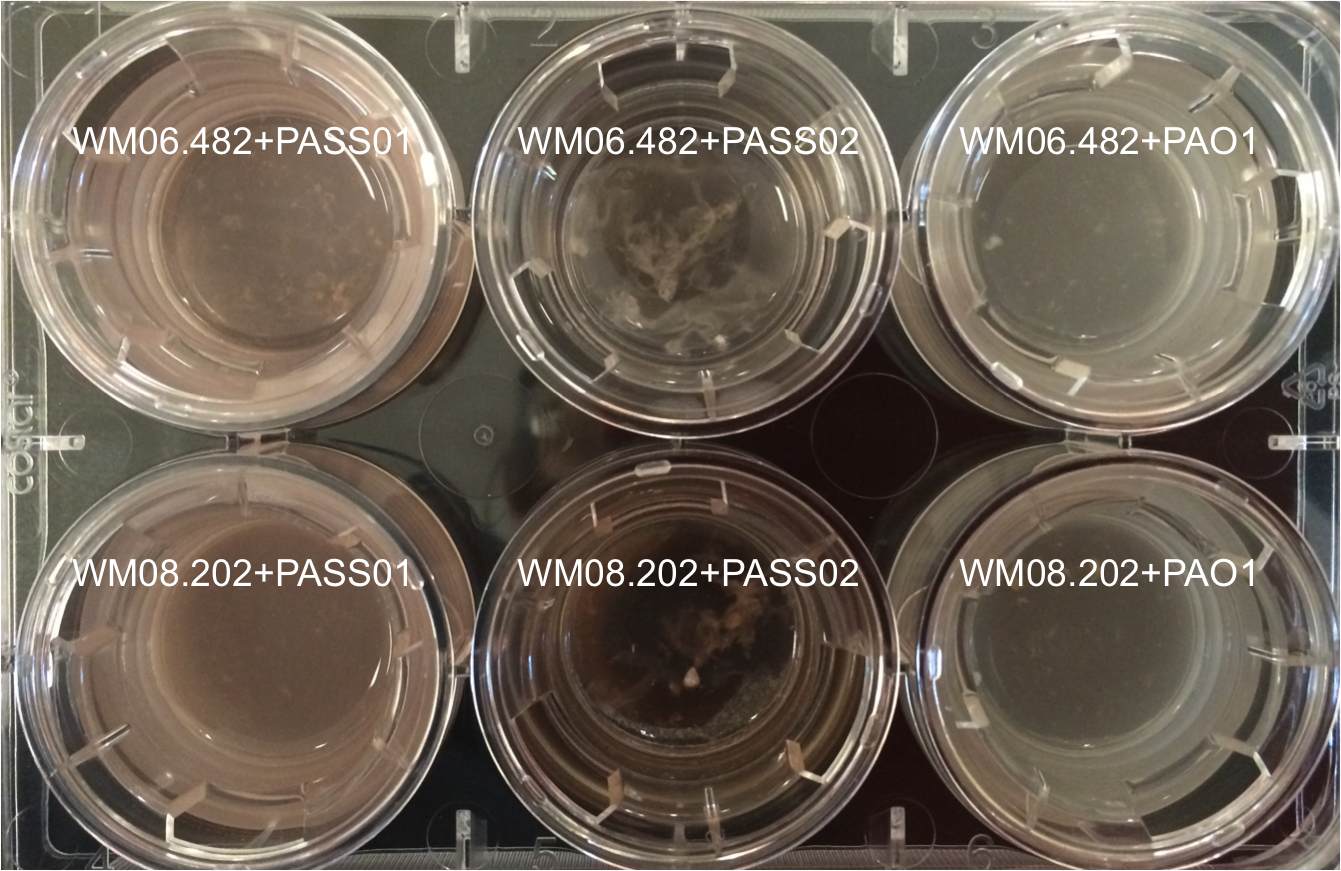
**

**Supplementary Figure 2.** **Transwell plate showing the formation of red pigment in the co-cultures involving different strains of *S. aurantiacum* (WM06.482 and WM 08.202) and *P. aeruginosa* (PASS01, PASS02 and PAO1).**
