# Supplementary figures and images for: Pseudomonas aeruginosa inhibits the growth of Scedosporium aurantiacum, an opportunistic fungal pathogen isolated from the lungs of cystic fibrosis patients
Source: Front Microbiol. 2015 Aug 24;6:866. doi: 10.3389/fmicb.2015.00866 (PMC4547459; doi:10.3389/fmicb.2015.00866)

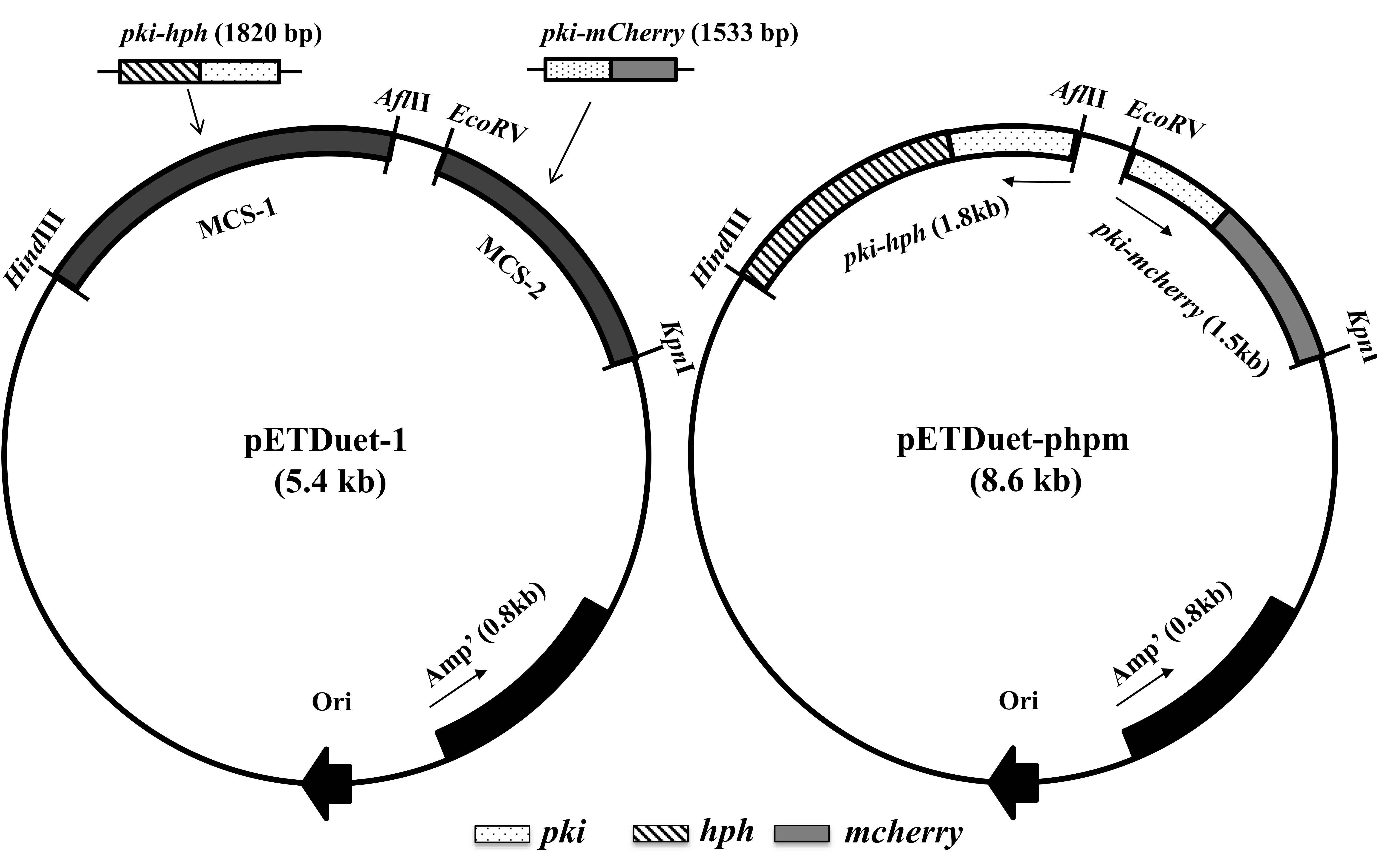

Supplement: Supplementary file 2 [file Image_1.TIFF]

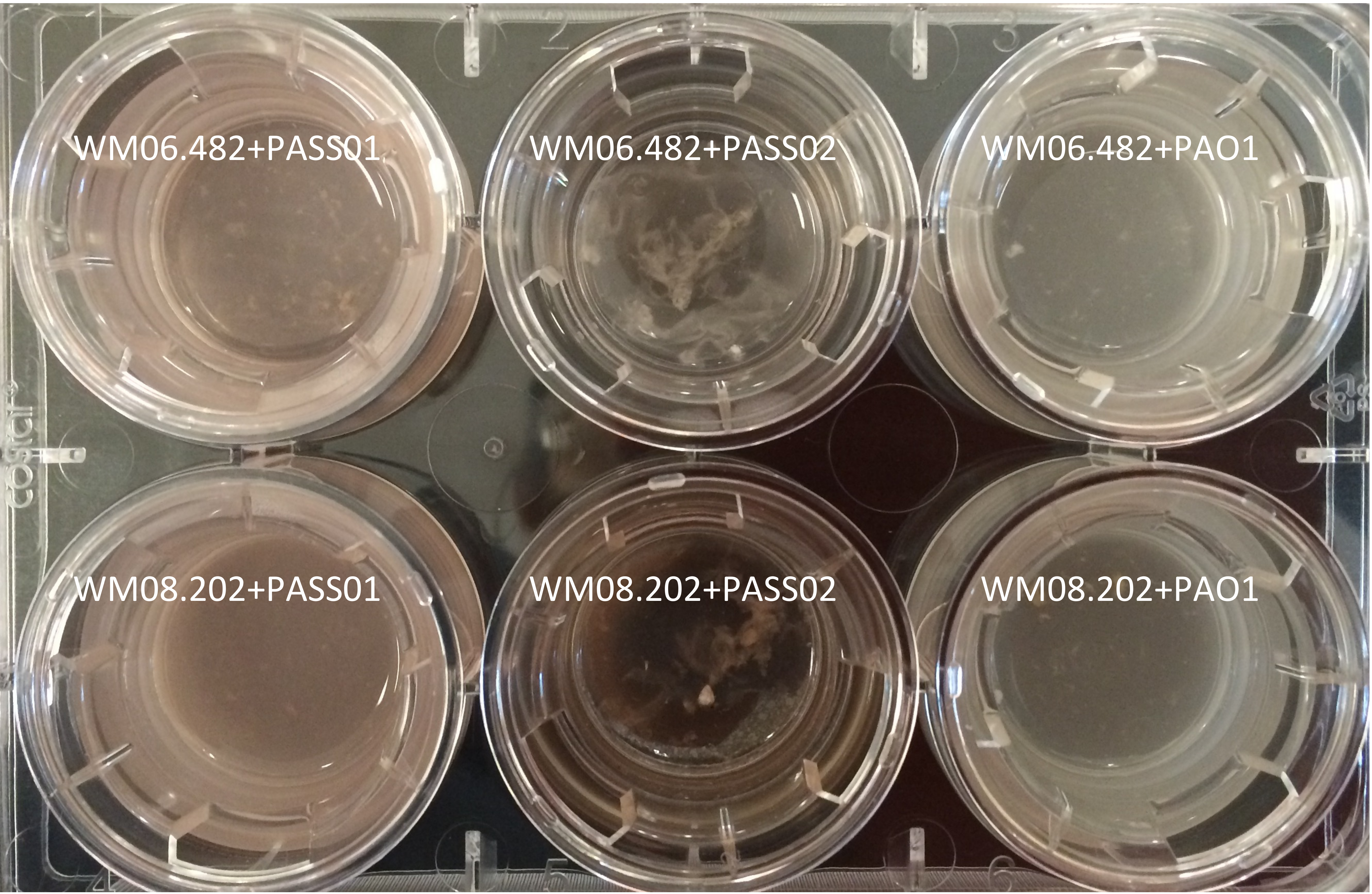

Supplement: Supplementary file 3 [file Image_2.TIFF]
